# Cardiopulmonary failure in children infected with Enterovirus A71

**DOI:** 10.1186/s12929-020-00650-1

**Published:** 2020-04-16

**Authors:** Shao-Hsuan Hsia, Jainn-Jim Lin, Oi-Wa Chan, Tzou-Yien Lin

**Affiliations:** 1 Division of Pediatric Critical Care Medicine, Department of Pediatrics, Chang Gung Memorial Hospital at Linkou, College of Medicine, Chang Gung University, Taoyuan, Taiwan; 2 Department of Pediatric Respiratory Therapy, Chang Gung Memorial Hospital at Linkou, College of Medicine, Chang Gung University, Taoyuan, Taiwan; 3 Department of Pediatrics, Chang Gung Memorial Hospital at Linkou, College of Medicine, Chang Gung University, No. 5, Fuxing Street, Guishan District, Taoyuan, 333 Taiwan

**Keywords:** Enterovirus A71, Pulmonary edema, Brainstem encephalitis, Cardiopulmonary failure, Children

## Abstract

Enterovirus A71 (EV-A71) is one of the causative pathogens of hand, foot, and mouth disease (HFMD), which may cause severe neurological and cardiopulmonary complications in children. In this review, we discuss the pathogenesis, clinical manifestations, management strategy, and clinical outcomes of cardiopulmonary failure (CPF) in patients with EV-A71 infection.

The pathogenesis of CPF involves both catecholamine-related cardiotoxicity following brainstem encephalitis and vasodilatory shock due to cytokine storm. Sympathetic hyperactivity, including tachycardia and hypertension, are the early clinical manifestations of cardiopulmonary involvement, which may progress to pulmonary edema/hemorrhage and/or CPF. The management strategy comprises multidisciplinary supportive treatment, including fluid management, positive pressure ventilation support, and use of milrinone, vasopressors, and inotropes. Some patients may require extracorporeal membrane oxygenation. Major neurological sequelae are almost inevitable once a child develops life-threatening illness. Long-term care of these children is an important medico-social issue.

## Background

Enterovirus A71 (EV-A71) was first described in 1969 [1]. Children infected with EV-A71 usually present with a self-limiting hand, foot, and mouth disease (HFMD); a majority of these patients show complete recovery. However, some children may develop severe systemic complications. Outbreaks of HFMD associated with high mortality rates have been reported across the world; however, a majority of these have occurred in the Asia–Pacific region. The disorder is characterized by involvement of three major organ systems, i.e., central nervous system (CNS), respiratory system, and cardiovascular system. The severity of involvement of these systems shows a close correlation with clinical outcomes [2–8]. We reviewed articles pertaining to the pathogenesis, clinical manifestations, management, and outcomes of cardiopulmonary failure (CPF) in children with EV-A71 infection. We also highlight the general care principles, respiratory therapy, and cardiovascular support for CPF and provide practical considerations.

## Pathogenesis of cardiopulmonary failure

The development of brainstem encephalitis (BE) represents a critical point in EV-A71-associated HFMD. Children with BE can subsequently develop autonomic nervous system (ANS) dysregulation, pulmonary edema (PE), heart failure, and CPF.

### Pulmonary edema

Several mechanisms underlying the development of PE in patients with BE have been proposed. Significant brainstem insult induces enhanced secretion of catecholamines (especially norepinephrine) from the sympathetic nerve endings; this causes peripheral vasoconstriction, pulmonary venoconstriction, and reduced left ventricular compliance, which subsequently augments the intrathoracic blood volume with excessive hydrostatic pressure [9, 10]. The second mechanism involves the release of some neurotransmitters (including neuropeptide Y) from the sympathetic nerves, which is linked to increased pulmonary capillary permeability [11]. The third mechanism involves extensive peripheral and CNS inflammatory responses, with abnormal production of cytokines [interleukin (IL)-10, IL-13, and interferon-gamma (IFN-g)] and depleted lymphocyte production, which increases endothelial permeability [12]. A complex interplay between all the three mechanisms may be involved in the pathogenesis of PE. There seems to be a decline in the incidence of PE/hemorrhage after widespread adoption of stage-based management in Taiwan [8].

### Heart failure

Myocardial injury and decreased contractility subsequently occur due to neurogenic stunned myocardium (NSM) [13] or norepinephrine cardiotoxicity [14]. Pathological findings of heart muscle in patients who died from EV-A71 infection were quite similar to those in cats with myocardial injury who were administered excessive norepinephrine.

In another study involving hemodynamic monitoring using pulmonary artery catheterization (PAC), two of the five patients initially showed elevation of pulmonary artery occlusion pressure (PAOP) to > 18 mmHg (maximum PAOP: 22 and 25 mmHg in the two patients); however, the PAOP eventually returned to ≤18 mmHg (in 24 and 40 h, respectively) in both patients. This suggested that intrathoracic hypervolemia and excessive hydrostatic pressure may not persist throughout the CPF stage. Notably, the systemic vascular resistance index (SVRI) of three of the five patients exceeded 1400 dynes·sec/cm^5^/m^2^ on at least one occasion; in addition, all four patients who underwent PAC monitoring for > 24 h showed decreases in SVRI to ≤1000 dynes·sec/cm^5^/m^2^ at some point. This indirectly suggests that the vasoconstrictive effect of the excessive catecholamine secretion resolves in the later part of the CPF stage, resulting in vasodilatory shock [15].

Therefore, the combined effect of NSM and vasodilatory shock may be the main cause of heart failure and mortality in patients with EV-A71 infection.

## Clinical manifestations

As listed in the World Health Organization (WHO) Guide to Clinical Management for HFMD 2011 [16], most patients with severe disease exhibit serial changes in the involved organ systems. The disease course is characterized by progression through four clinical stages: 1) HFMD/herpangina; 2) CNS involvement; 3) ANS dysregulation; and 4) CPF. Although “cardiopulmonary failure” is considered as the final stage, the heart and lungs may be involved at a much earlier stage, possibly between CNS involvement and ANS dysregulation (Table 1). Therefore, concerted efforts to prevent CPF should be initiated from the time of appearance of the earliest signs of heart and lung involvement, such as prehypertension, tachycardia, tachypnea, and decreasing PaO_2_/fraction of inspired oxygen (FiO_2_). It is recommended that patients are managed in a pediatric intensive care unit.

**Table 1:** Clinical manifestations of EV-A71 infections: four stages and management

Stage	Signs of cardiopulmonary involvement	Other clinical manifestations	Laboratory/exam findings	Management of CPF
1. HFMD/ Herpangina	➢ None	➢ Vesicular rash on hands, elbows, feet, knees and buttocks ➢ Oral ulcers and herpangina ➢ Fever ➢ GI symptoms	➢ Generally within normal limit	➢ None
2. CNS Involvement	➢ Prehypertension (systolic pressure between 90 and 95% by age ≈ 105–115 mmHg for high risk age groups) [17] ➢ Tachycardia (resting heart rate higher than normal but < 150/min) and abnormal heart rate variability( [18] [19];)	➢ Myoclonic jerk ➢ Meningism ➢ Ataxia, tremors ➢ Lethargy ➢ Limb weakness and Polio-like syndrome ➢ Altered mental status ➢ Generalized tonic-clonic convulsion	➢ CSF pleocytosis ➢ MRI: high signal intensities on T2 weighted images in brainstem and spinal cord [20]	➢ Limited fluid replacement therapy ➢ Early intubation and PPV ➢ IVIg
3. ANS Dysregulation	➢ Inappropriate tachycardia (resting heart rate > 150/min) ➢ Severe hypertension (systolic pressure > 95% by age ≈ 115–120 mmHg for high risk age groups) [17] ➢ Tachypnea ➢ Hemoptysis, pink frothy sputum ➢ Low Pao2: Fio2 ratio ➢ Chest radiography: PE/H	➢ Hyperglycemia (> 150 mg/dL) ➢ Profuse sweating ➢ Cranial nerve abnormality and GCS deterioration	➢ Hypoxemia ➢ Hyperglycemia	➢ Intubation and PPV ➢ HFOV ➢ Milrinone
4. CPF	➢ Hypotension ➢ Low cardiac output ➢ Signs of poor perfusion ➢ Absence of spontaneous respiration though pulmonary edema improves	➢ Coma, paralysis ➢ Neurological sequelae	➢ Elevated troponin I and cardiac enzyme ➢ Lactic acidosis ➢ Poor left ventricle ejection fraction	➢ Dopamine and epinephrine ➢ ECMO ➢ Volume expansion

## Management

### General management

Early identification of signs of deterioration is essential. Any patient with herpangina/HFMD who exhibits signs and symptoms of CNS involvement, such as frequent myoclonic jerks, limb weakness, seizure, ataxia, cranial nerve abnormality, or significant lethargy, should be subjected to close monitoring of cardiopulmonary function. In the early stages of cardiovascular deterioration, patients usually present with tachycardia and cold extremities, which may mimic the symptoms of hypovolemia. However, high-volume intravenous expansion during this period should be avoided as it may induce or aggravate pulmonary edema [16].

Intravenous immunoglobulin (IVIg) therapy is based on the assumption that the pooled immunoglobulins may neutralize the enterovirus, similar to that in neonatal enterovirus sepsis [21]. In addition, IVIg therapy may have an immunomodulatory effect in patients with proinflammatory cytokines [22, 23]. IVIg therapy is recommended for patients with CNS involvement [16]. However, if this therapy is not administered when patients progress to ANS dysregulation and CPF, it should be administered carefully as the large fluid volume may aggravate CPF.

### Respiratory therapy

Respiratory failure is caused by PE/hemorrhage and apnea during ANS dysregulation. It is a serious condition that may rapidly progress to the CPF stage. In patients with fulminant disease course, death may occur rapidly after sudden and severe hemoptysis [2].

Endotracheal intubation and positive pressure ventilation (PPV) may be considered in the following conditions: rapid deterioration of mental status [Glasgow coma scale (GCS) < 9]; severe hypoxemia requiring a high FiO_2_; inability to maintain airway patency, such as frequent choking by saliva; apnea; and heart failure. A higher positive end-expiratory pressure of 6–8 cmH_2_O is usually required to maintain oxygenation and prevent atelectasis [24].

High frequency oscillatory ventilation should be considered if very high FiO_2_ and mean airway pressure (P$\overline{aw}$) are required to maintain oxygenation, as suggested in acute respiratory distress syndrome [25].

### Cardiovascular support

Milrinone is a phosphodiesterase 3 inhibitor that promotes cardiac contractility and decreases both pulmonary and systemic vascular resistance [26]. Milrinone has also been shown to exhibit an anti-inflammatory effect [27]. In a historical controlled case series, the milrinone-treated group had lower mortality, decreased sympathetic tachycardia, and marked decrease in IL-13 level [28]. In a randomized controlled trial, the milrinone treatment group showed lower 1-week mortality and longer median duration of ventilator-free period than the control group [29]. Early initiation of milrinone therapy is recommended in patients who show echocardiographic evidence of impaired heart function, even if the blood pressure and organ perfusion are at an acceptable level [24, 30].

Excessive catecholamines may induce PE, cause myocardial damage, and augment virus infection; therefore, WHO guidelines do not recommend the use of dopamine, epinephrine, or norepinephrine [13, 16, 31, 32]. However, the adverse effects of catecholamines are mainly attributable to their α1-adrenergic effect [33] (which occurs after the use of norepinephrine and at high dopamine, epinephrine, and dobutamine infusion rates), and application of extracorporeal membrane oxygenation (ECMO) in infants and toddlers can cause severe complications [34]. Therefore, prior to considering ECMO, low–intermediate doses of dopamine (≤10 mcg/kg/min) and epinephrine (≤0.1 mcg/kg/min) may be administered in high-risk patients if the blood pressure decreased to below the normal range (approximately 85–90 mmHg systolic pressure) [35].

ECMO is the last rescue treatment for refractory CPF. In a retrospective study of 13 children (mean age, 16 ± 10 months) with EV-A71 CPF, the clinical manifestations and outcomes (2000–2008, present cohort) were compared with those of 10 other children (1998–2000, past cohort). The present cohort showed significantly better neurological outcomes (46% vs. 0%, *P* = 0.005) and a significantly higher survival rate (77% vs. 30%, *P* = 0.024) compared with the past cohort [36]. Unfortunately, some of the survivors had mild-to-severe neurological sequelae. ECMO can be considered for patients with refractory hypotension, those with signs of poor end-organ perfusion (such as profound lactic acidosis and oliguria) or poor left ventricle contractility (on echocardiography or hemodynamic monitoring), and those who require high doses of inotropes [16, 24].

**Fig. 1:**
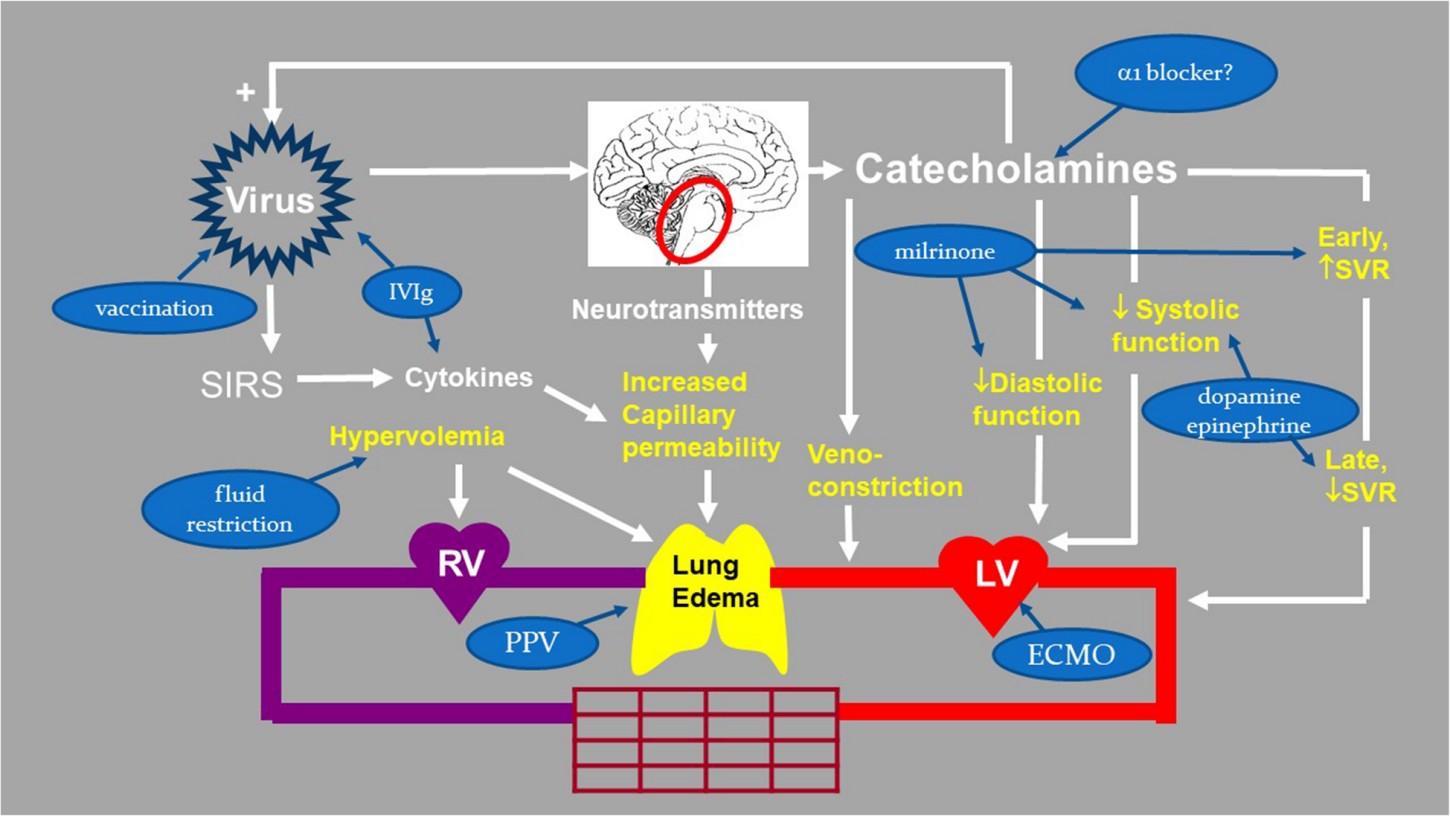
Schematic illustration of the pathogenesis and the available management options for EV-A71-associated cardiopulmonary failure. Text in white font indicates the direct causes of cardiopulmonary failure. Text in yellow font indicates the mechanisms. Text within blue circles indicates the specific management options for CPF. RV: right ventricle; LV: left ventricle

Other suggested treatments include α and β blockers, vasodilators, and diuretics [24]. In a rat model of PE, prazosin (α1 receptor blocker) was shown to preserve cardiac output, reverse neutrophil infiltration in the lungs, and prevent pulmonary hemorrhagic edema; however, those treated with propranolol (a β-receptor blocker) died within 2 h 30 min. A case report also documented successful treatment of PE with phentolamine in a human [37]. No trials of vasodilators and diuretics in this setting have been conducted; therefore, current guidelines do not recommend their routine use. The present understanding of the pathogenetic mechanisms and the current management options for EV-A71-associated CPF are illustrated in Fig. 1. First, the virus induces a systemic inflammatory response and causes BE. Second, BE induces excessive secretion of catecholamines, followed by decreased left ventricular myocardial contractility, diastolic dysfunction, and peripheral vasoconstriction. Third, these three effects combined with neurotransmitter-induced changes in pulmonary capillary permeability and fluid overload cause PE/hemorrhage. Furthermore, catecholamine therapy (especially norepinephrine) may enhance the viral activity and the inflammatory response. Accordingly, vaccines (rapid progress has been made in the development of EV-A71 vaccines [38]) can be used to prevent virus infection, IVIg can be used to reduce systemic inflammatory response, fluid overload can be avoided, milrinone can be administered at an early stage to prevent pulmonary vascular congestion, PPV can be used to prevent abnormal alveolar gas exchange resulting from PE/hemorrhage, vasopressors can be used when necessary, and timely use of ECMO can be considered as a life-saving measure.

## Outcome

In a long-term follow up study, of 28 patients who survived CPF, 18 (64%) had limb weakness and atrophy, 17 (61%) required tube feeding, and 16 (57%) required ventilator support. In addition, 21 of 28 patients (75%) exhibited delayed neurodevelopment as assessed using the Denver Developmental Screening Test (DDST II) [39]. These survivors require long-term medical care, socioeconomic assistance, and educational support.

## Conclusions

During the last 20 years, we have progressed from ignorance to a better understanding of EV-A71-caused HFMD. CPF is characterized by PE, NSM, and vasodilatory shock. There is no commercially available antiviral drug against EV71 to prevent viral replication or prevent CPF. IVIg therapy, milrinone administration, and stage-based management appear to be associated with better outcomes. Identifying the signs of disease progression, anticipating the development of CPF, and providing appropriate multidisciplinary supportive treatment are key tenets of management. ECMO seems to be the last rescue approach for critically ill children with refractory CPF.

## Data Availability

Not applicable.

## Abbreviations

ANS: Autonomic nervous system
BE: Brainstem encephalitis
CNS: Central nervous system
CPF: Cardiopulmonary failure
ECMO: Extracorporeal membrane oxygenation
EV-A71: Enterovirus A71
FiO_2_: Fraction of inspired oxygen
GCS: Glasgow coma scale
HFMD: Hand, foot, and mouth disease
IVIg: Intravenous immunoglobulin
NSM: Neurogenic stunned myocardium
PE/H: Pulmonary edema/hemorrhage
PAC: Pulmonary artery catheterization
PAOP: Pulmonary artery occlusion pressure
P$\overline{aw}$: Mean airway pressure
PPV: Positive pressure ventilation
SVRI: Systemic vascular resistance index
WHO: World Health Organization
